# Computed Tomography Urography: State of the Art and Beyond

**DOI:** 10.3390/tomography9030075

**Published:** 2023-04-30

**Authors:** Michaela Cellina, Maurizio Cè, Nicolo’ Rossini, Laura Maria Cacioppa, Velio Ascenti, Gianpaolo Carrafiello, Chiara Floridi

**Affiliations:** 1 Radiology Department, Fatebenefratelli Hospital, ASST Fatebenefratelli Sacco, Piazza Principessa Clotilde 3, 20121 Milan, Italy; 2 Postgraduation School in Radiodiagnostics, Università degli Studi di Milano, Via Festa del Perdono 7, 20122 Milan, Italy; 3 Department of Clinical, Special and Dental Sciences, University Politecnica delle Marche, 60126 Ancona, Italy; 4 Division of Interventional Radiology, Department of Radiological Sciences, University Politecnica delle Marche, 60126 Ancona, Italy; 5 Radiology Department, Policlinico di Milano Ospedale Maggiore|Fondazione IRCCS Ca’ Granda, Via Francesco Sforza 35, 20122 Milan, Italy; 6 Division of Special and Pediatric Radiology, Department of Radiology, University Hospital “Umberto I-Lancisi-Salesi”, 60126 Ancona, Italy

**Keywords:** CT urography, renal cancer imaging, artificial intelligence, Dual-Energy Computed Tomography, AI-based reconstruction algorithms, Computed Tomography

## Abstract

Computed Tomography Urography (CTU) is a multiphase CT examination optimized for imaging kidneys, ureters, and bladder, complemented by post-contrast excretory phase imaging. Different protocols are available for contrast administration and image acquisition and timing, with different strengths and limits, mainly related to kidney enhancement, ureters distension and opacification, and radiation exposure. The availability of new reconstruction algorithms, such as iterative and deep-learning-based reconstruction has dramatically improved the image quality and reducing radiation exposure at the same time. Dual-Energy Computed Tomography also has an important role in this type of examination, with the possibility of renal stone characterization, the availability of synthetic unenhanced phases to reduce radiation dose, and the availability of iodine maps for a better interpretation of renal masses. We also describe the new artificial intelligence applications for CTU, focusing on radiomics to predict tumor grading and patients’ outcome for a personalized therapeutic approach. In this narrative review, we provide a comprehensive overview of CTU from the traditional to the newest acquisition techniques and reconstruction algorithms, and the possibility of advanced imaging interpretation to provide an up-to-date guide for radiologists who want to better comprehend this technique.

## 1. Introduction

Computed Tomography Urography (CTU) is defined as an abdominal multiphase CT examination optimized for imaging kidneys, ureter, and bladder, including post-contrast excretory phase imaging [1].

Over the past decade, CTU has become the primary imaging technique for evaluating the urinary tract and is widely accepted as part of the routine evaluation of patients with hematuria [2].

According to the 2019 American College of Radiology Appropriateness Criteria, CTU is recommended as the first-line imaging method in patients with microhematuria and risk factors for urologic malignancy [3]. The American Urological Association recommends its execution in patients with asymptomatic microhematuria persisting after therapy and for the exclusion of any benign etiologies [4]. These indications have been also confirmed by the American College of Physicians [5]. The main indications for CTU are listed in Table 1 [6,7].

The usefulness of CTU as a diagnostic tool is connected to its optimized acquisition technique. The principal aim is to achieve optimal distension and opacification of the upper tract collecting system, ureters, and urinary bladder in the excretory phase, [7]; the other aim is to fit the “As Low As Reasonably Achievable” principle [8], obtaining an adequate image quality while limiting radiation exposure [9].

There is no consensus on a standard protocol or national and institutional guidelines, and CTU is still performed with widely different acquisition and contrast administration protocols [10,11].

The most widely used technique consists of three post-contrastographic phases including corticomedullary (CMP), nephrographic, and excretory phases.

The unenhanced phase enables stone detection and helps the characterization of renal masses, as it allows the differentiation of non-enhanced to enhance lesions (<10 Hounsfield Unites, HU, of increase from non-contrast to post-contrast series is considered non-enhancing, 10–20 HU of increase is indeterminate, >20 HU of the increase represents a significant enhancement) [12].

**Figure 1:**
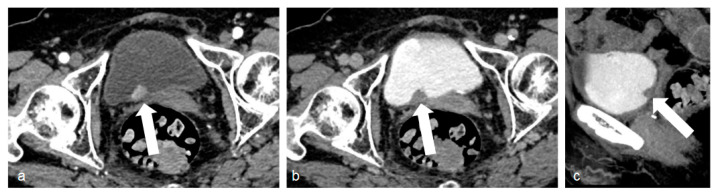
Images of bladder cancer. (**a**) The corticomedullary phase acquisition with a polypoid lesion originating from the posterior bladder wall, characterized by high contrast enhancement (white arrow). In the excretory phase (**b**) and its sagittal reconstruction (**c**), the lesion is clearly visible as a filling defect in the iodinated urine-filled bladder (white arrows).

**Figure 2:**
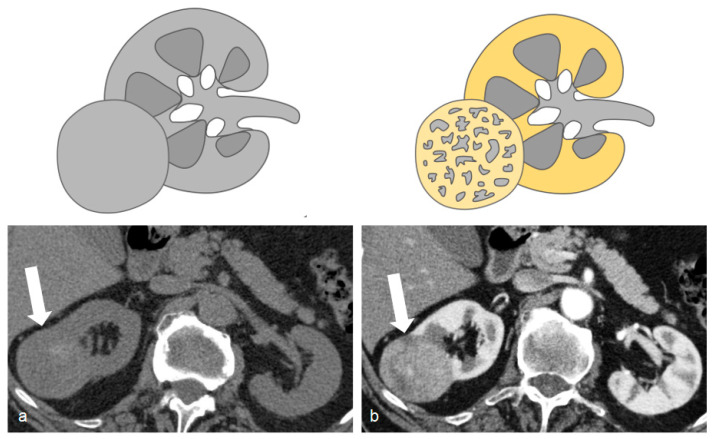
Unenhanced (**a**) and arterial post-contrastographic phase (**b**) showing the presence of a right clear cell carcinoma (white arrows), isodense compared to the renal parenchyma in the unenhanced phase (**a**), and characterized by vivid contrast enhancement in the arterial phase (**b**).

CMP is usually acquired 25 s after reaching 200 HU in the region of interest in the abdominal aorta and has the highest sensitivity (95%) and negative predictive value (99%) for bladder cancer [13] (Figure 1) and renal cell carcinoma detection [7] (Figure 2).

The nephrographic and excretory phases can be set at 100 s and 10–15 min after contrast medium injection, respectively [14].

Some studies demonstrated the superiority of the nephrographic phase in the identification of urothelial carcinoma when compared to the excretory phase [14,15,16], whereas the excretory phase has traditionally been considered the most valuable phase for the identification of urothelial carcinomas [14,17,18].

This study aims to provide an overview of all CTU acquisition protocols, ancillary techniques for exam implementation, and reconstruction algorithms. The principal available novelties such as Dual-Energy CT acquisition and artificial intelligence applications are also addressed. A good understanding of basic and advanced acquisition and post-processing techniques may help radiologists in identifying the best acquisition protocols and available technology for each purpose.

## 2. Acquisition Technique

Different protocols of contrast administration are available and listed in Table 2.

### 2.1. Single Bolus

**Figure 3:**
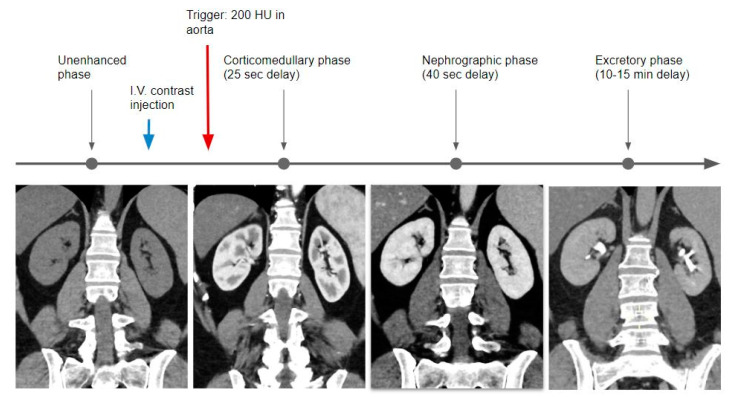
Graphical representation of the single bolus acquisition technique.

The traditional study technique includes the acquisition of a non-contrast phase, followed by the injection of the full dose of contrast medium and acquisition of the nephrographic (80 to 120 s) and delayed excretory phases (5 to 15 min); additional acquisition of the CMP (30 to 40 s) is optional [18] (Table 2) (Figure 3).

The first acquired phase is the CMP, usually acquired with a bolus tracking technique, placing a region of interest in the abdominal aorta, with a threshold of 200 HU, with an estimated acquisition delay set at 25 s. In this phase, the kidney contrast enhancement is related to the arterial inflow. The nephrographic phase is then acquired at a delay of about 40 s when the renal parenchyma is almost homogeneous in density.

In the excretory phase, the renal parenchyma is homogeneous but characterized by a markedly reduced density in comparison with the previous phases, with the calyces and pelvis filled with iodinated urine.

This single bolus technique allows maximal opacification and distension of the urinary tract as the whole contrast volume contributes to the nephrographic and excretory phases [7,19]. This protocol is also described as the most sensitive for renal cell and transitional cell carcinomas. Furthermore, including unenhanced images maximizes its sensitivity in detecting renal and ureteral stones [7]. However, since three or four distinct acquisitions are performed, this technique results in the highest ionizing dose [19].

Due to the increase in radiation exposure, there is no consensus about the need for CMP acquisition. The advantages of the CMP are precise vascular and perfusion information [20], a better characterization of renal cortical masses [21], and the detection of hypervascular metastases. CMP is also characterized by a higher sensitivity and negative predictive value for the detection of bladder tumors than either the nephrographic or excretory phases alone [22,23]. Many radiologists, however, omit this phase because the small added benefit does not justify the increased dose [24].

The acquisition timing can vary for each acquisition phase, particularly for the nephrographic and delayed excretory phases. The nephrographic phase is usually performed between 80 and 120 s post-contrast injection [25]. Images acquired too early, before the cortex and medulla are uniformly opacified, can limit image interpretation, whereas images acquired too late can detect the early excretion of contrast into the collecting system, hiding mucosal enhancement within the renal pelvis.

The excretory phase begins at 3 min post-contrast administration [25] with timing delays of up to 15 min reported in the literature [26].

A timing delay should be considered in patients with impaired renal function and known dilatation of the excretory system: in these cases, an excretory phase performed in a prone position can help the distension and opacification of the urinary tract.

To avoid acquiring images too early, some authors proposed the use of a single-slice, low-dose test image at the mid-ureter level to verify the opacification and confirm the timing of the excretory phase imaging [27], but this increases the complexity of the protocol.

An excessive delay can increase the density of contrast within the collecting system, resulting in difficulties in the identification of subtle filling defects through the dense contrast [7] and in beam hardening artifacts in the bladder [28].

Some authors proposed the acquisition of a phase intermediate between the CMP and nephrographic phases, at 60–70 s post contrast medium injection, called the urothelial phase, which demonstrated a high detection rate for upper tract urothelial lesions, with a sensitivity and specificity of 95% and 97%, respectively [29], and higher sensitivity for the detection of bladder tumors than the excretory phase alone (89.3% vs. 70.5%) [15]. However, this phase has not yet been proven to be superior either to the nephrographic phase or the combined nephrographic and excretory phases if used alone, and its addition to the existing phases would result in an unjustifiable increased radiation dose in the absence of supporting evidence of a clear added benefit.

The single bolus technique is the simplest to perform for technologists due to the need for just a single contrast administration, at least partially accounting for the diffusion of this technique.

### 2.2. Split Bolus

The split bolus protocol is recommended for lower radiation exposure [10].

**Figure 4:**
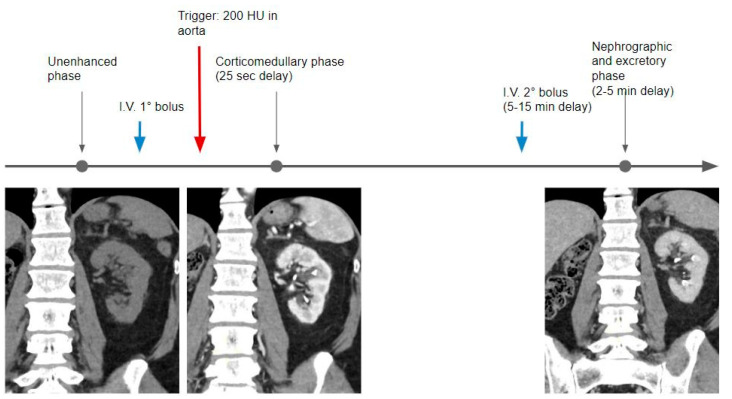
In the split bolus technique, after the unenhanced phase, the first portion of the whole contrast medium dose is administered (usually one-third or half); then, using the bolus tracking technique and a delay of 25 s, the corticomedullary phase is acquired. A second bolus of contrast medium is injected at a variable timing (5–10 min of delay), followed by a third CT acquisition, where the kidneys are in the nephrographic phase and the calyces and pelvis are filled with iodinated urine.

In the split bolus technique, the nephrographic (for the detection of renal masses) and excretory phases (for urothelial neoplasms) are acquired at the same time, avoiding one acquisition, and thereby reducing the radiation dose by approximately one-third (Figure 4).

In this protocol, the contrast bolus is administered in two separate injections. After the unenhanced phase, the first part (usually one-third or half) of the contrast is injected and the optional CMP is obtained, then the second part of the contrast (usually a dose larger than or equal to the first one), is injected about 5–10 min later, and combined nephrographic–excretory phase images are acquired at 2–5 min when the kidneys show enhancement of the renal parenchyma and opacification of the collecting systems occurs [7] (Table 2).

The administration of contrast varies widely among different studies with different ratios between the first and second portions of the split bolus, as well as the optimal delay time [7,30,31].

Some authors suggested the use of a larger bolus as a second injection to improve renal parenchymal enhancement and a delay time of 8 min from the first injection to maximize ureteral distension and opacification [30]. Others prefer the second dose injection 10 min after the first bolus, with a combined nephrographic and excretory phase acquisition at 700 s after the beginning of the first contrast media injection [31].

The advantage of this technique is the combination of two separate contrast phases (nephrographic and excretory phases) into a single acquisition, thereby reducing the total number of images acquired and, accordingly, the total radiation exposure, which is important in younger patients.

The main disadvantage encountered is a lower contribution of contrast medium to kidney enhancement and to distension and opacification of the urinary collecting system, which may reduce image quality and sensitivity for the detection of small renal cell carcinomas [19] and subtle transitional cell carcinomas [7].

### 2.3. Triple Bolus

**Figure 5:**
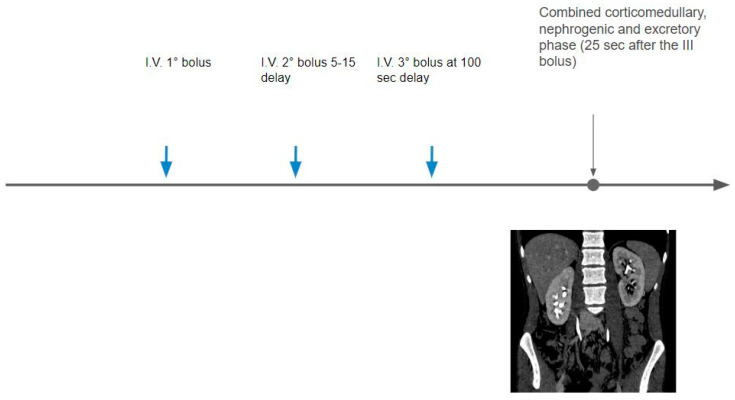
The contrast medium dose is divided into three parts: the first one is administrated after the non-contrast acquisition; the second bolus is injected after a variable time delay (5–15 min); and then a third bolus at 100 s of delay, followed by the acquisition of a mixed corticomedullary and excretory phase.

The triple bolus technique is performed in a few institutions and is based on the separation of the total contrast volume into three injections. An optional unenhanced phase can be acquired, the first bolus part is then injected, followed by a delay time, the second bolus part is then injected, a delay time elapses, then the third portion of the bolus is administered, and finally, post-contrast images are acquired. The resulting acquisition combines CMP, nephrographic, and excretory phases, allowing simultaneous arterial, parenchymal, and collecting system enhancement (Figure 5).

This protocol significantly decreases the total radiation dose because of the reduction in the total number of acquired contrast phases; however, due to the bolus splitting, only a portion of the total contrast medium volume contributes to excretory imaging, thus resulting in potential limited distension and opacification of the ureters.

This protocol also provides limited accuracy in renal cell carcinoma detection, due to the absence of a dedicated arterial phase image acquisition, which is the most sensitive to clear cell renal cell carcinomas [7].

### 2.4. Attempts to Optimize the Excretory Phase

To achieve adequate distension and whole opacification of the urinary tract in a single excretory phase, ancillary techniques have been proposed, but none have been universally adopted in practice.

These techniques include oral or intravenous hydration before the acquisition; intravenous furosemide administered before the intravenous contrast material; use of abdominal compression devices (belts); prone patient positioning; and, if images of the excretory phase are suboptimal, additional delayed phase imaging [19,32].

Hydration has a role in improving excretory system distension and contrast dilution and is usually performed with the administration of 100–250 mL of saline solution intravenously before the study, or with oral administration of 400 mL of water before the study [33,34].

The administration of a diuretic, usually intravenous furosemide, has also been reported to increase the urine flow rate and enhance urinary tract opacification and distension [35,36]. Moreover, the diuretic promotes contrast dilution in the collecting system, allowing the detection of subtle urothelial thickenings through the dense contrast [7].

A recent study supported the use of 5 mg of furosemide to achieve optimal bladder filling in CTU to increase the identification of tumors [18]. However, the use of diuretics is not widespread due to the need for extra time and personnel for the administration and investigation of patients’ medication allergies and contraindications, resulting in a more difficult workflow.

The use of other ancillary techniques is less supported by the literature; in particular, no evidence supports the use of a compressive belt or acquisition in the prone position in the improvement of ureteral distension and opacification, and these techniques risk resulting in increased complexity of the technique, prolonged examination time, and additional acquisitions with increased radiation exposure [7].

Some institutions have also modified the volume of contrast material and the saline solution administration to maximize ureteral distension; for example, the use of a larger volume of more dilute contrast has been proposed to increase excretion into the collecting system [18].

No consensus has been reached about these attempts to optimize the excretory phase.

## 3. Image Reconstruction and Post-Processing

### 3.1. Iterative Reconstruction (IR)

The number of CTU phases varies between two and four, and the effective dose can reach 25–35 mSv, especially when using dated equipment, depending on the performed phases and acquisition parameters [37]. As the cancer risk has a linear correlation with radiation exposure without a threshold, even a small amount of radiation dose can contribute to increasing the risk. Due to the multi-phasic characteristic of CTU, the patient’s lifetime cancer risk related to radiation exposure can be higher, especially in young patients; therefore, a substantial effort has been made to reduce the radiation exposure. Lowering the tube potential results in a significant reduction of the radiation dose [38] with increased visualization of the opacified urinary system in the excretory phase; the use of a reduced tube voltage (80 kV) to the excretory phase of CTU has been introduced in the last decade [39].

To generate diagnostically optimal images at a lower tube voltage, reconstruction algorithms different from filtered back projection should be applied.

IR applies a correction loop in the image reconstruction from the raw image data and shows the effectiveness of reducing radiation without any degradation of image quality, thanks to the reduction of the image noise, while still maintaining an optimal image quality [40,41]. Different IR algorithms are available from CT scanner manufacturers [42]. These algorithms primarily differ in their reconstruction methods.

Low tube voltage CT protocols with IR reduce the image noise and help in the identification of enhanced urothelial cells and decreases the attenuation of fat within renal lesions such as angiomyolipoma [43].

### 3.2. Deep Learning Image Reconstruction

Deep convolutional neural network-based models have been applied to low-dose CT examinations to imitate standard-dose filtered back projection (FBP) image texture while ensuring low image noise, streak artifacts suppression, increased low contrast lesion detectability, and high resolution [44].

**Figure 6:**
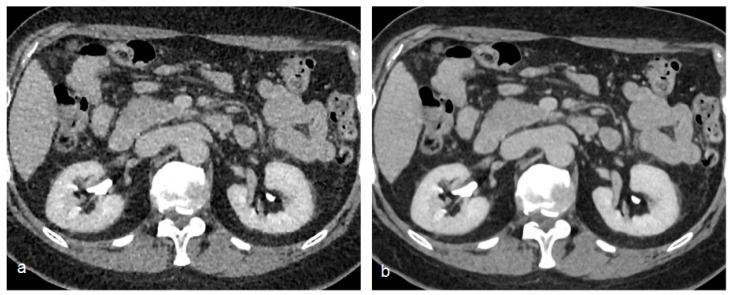
Comparison of a standard filtered back projection reconstruction (**a**) of an excretory phase with an artificial intelligence-based reconstruction algorithm (**b**) (AiCE—Advanced intelligent Clear-IQ Engine, Canon Medical Solutions). Note the significant noise reduction of the artificial intelligence-based reconstruction (**b**).

Phantom studies have demonstrated the ability of these new reconstruction algorithms both in lowering image noise and in improving spatial resolution with no increases in noise levels [45] (Figure 6).

### 3.3. Post-Processing

Although standard axial image analysis may be sufficient for the study of other organs in the abdomen and pelvis, the application of post-processing image techniques is beneficial for the evaluation of the collecting system and in the detection of subtle urothelial tumors.

Multiplanar, Maximum Intensity Projection (MIP), and tridimensional volume rendering reconstructions can be performed to increase sensitivity and visualization of the kidneys and urothelium.

**Figure 7:**
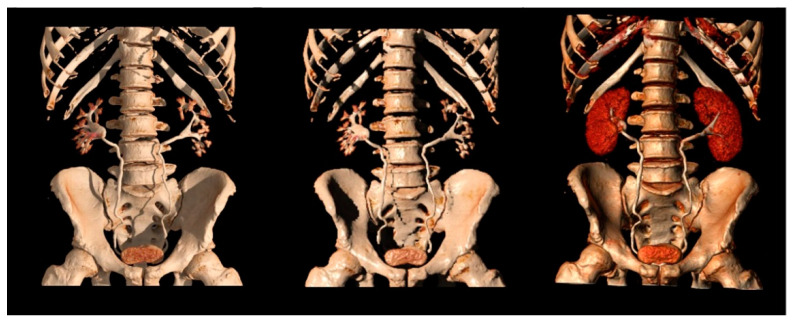
Different 3D reconstructions of the excretory phase.

Native thin (0.5–0.75 mm) axial images are usually submitted to coronal and sagittal reformations, then further to MIP and 3D reconstructions (Figure 7).

MIP reformations are constructed from the highest attenuation voxels in a dataset and projected into a 3D format. These reconstructions are particularly helpful in evaluating the collecting systems and ureters, allowing a complete and quick overview of the high-density contrast within the collecting systems, and highlighting subtle filling defects, focal thickening of the excretory system walls, luminal narrowing or strictures, calyceal abnormalities, hydronephrosis, and hydroureter.

Three-dimensional reconstructions provide specific colors to each voxel in a data set according to its attenuation and relationship to other adjacent voxels, allowing the visualization of the whole opacified excretory system. Some authors support their role in the identification of slight urothelial thickening, especially in cases of reduced excretion of the contrast into the collecting system, when MIP reformations are of limited usefulness [7,19,46].

## 4. Dual-Energy CT (DECT)

### 4.1. DECT Basic Concepts

DECT technology is based on the analysis of the attenuation spectra of materials at different energies, enabling material characterization [47].

Different CBCT technologies are available: dual source (two X-ray sources operating simultaneously at low and high tube potentials and two detectors capturing low- and high-energy spectra) [47], rapid switching (a single tube quickly switching from low to high energy in 0.4 ms, and a single fast-response spectral detector), sequential (single X-ray source switching between low and high energy after each rotation), and spectral detector (a single X-ray source and specialized detector with two scintillation layers capturing low- and high-energy photons) [48].

By examining the attenuation spectra of the same anatomical district at multiple energies, DECT provides attenuation characteristics of the structures. The interaction between the X-rays and scanned material, which is connected to the physical features of the material such as density and atomic number, determines the extent of change in attenuation between the low- and high-energy spectra [49].

Low-energy photons with high attenuation and high-energy photons with low attenuation characterize the X-ray beam intensity. Attenuation increases as the density and the atomic number of the material grows, while the energy of the X-ray beam decreases.

As a result, different materials have different attenuation curves related to both the material’s intrinsic properties and the energy of the beam employed.

Due to larger iodine attenuation coefficients at lower energy levels, low-energy pictures typically exhibit stronger contrast and enhanced lesion detection in post-contrast acquisitions; however, they are characterized by higher image noise [50].

Low-energy images emphasize iodine, whose density increases when the beam voltage is dropped, resulting in a larger contrast with the background tissues, improving the detection of contrast-enhancing lesions.

The principal aim of DECT protocols is to achieve high-contrast images with low noise by combining the stronger iodine attenuation of the low kVp spectrum through monoenergetic imaging with the lower image noise of the high kVp spectrum through post-processing noise-optimizing algorithms [51].

The high- and low-energy data from DECT acquisition can be used to create a single-energy CT-like image including attenuation values and structural information or to make other material-specific reconstructions [48].

### 4.2. DECT Virtual Non-Contrast Images

DECT has a large number of benefits. First, owing to decomposition analysis and the generation of virtual non-contrast CT images, which are obtained by separating iodine from soft tissue and water, DECT reduces the radiation exposure [52].

**Figure 8:**
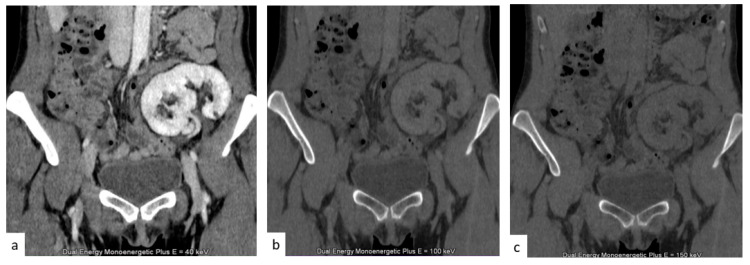
(**a**) Nephrographic phase of a transplanted kidney. (**b**,**c**) Two virtual non-contrast images at 100 (**b**) and 150 (**c**) KeV.

Virtual unenhanced images (Figure 8) enable the omission of the true unenhanced scans, with an up to 50% dose reduction, when applied to the split bolus contrast administration technique, while maintaining the diagnostic value of the exam [53].

Virtual non-contrast images allow the identification of calculi and hemorrhagic changes and help in the characterization of renal masses, but without the acquisition of a distinct unenhanced phase, thus limiting the radiation dose [54,55].

The sensitivity of stone detection of virtual non-contrast images reached 95% [56]; false negativity is the result of accidental subtraction of small, low attenuating stones surrounded by dense contrast urine [57,58].

False positives may be generated by the presence of dense contrast urine foci accumulated within the urinary tract and falsely recognized as urinary stones on virtual non-contrast reconstructions [59]. The dilution of contrast urine by oral hydration, furosemide, and lower contrast volumes in the first bolus, as well as using 100–140 kVp instead of 80–140 kVp pairs, increases the accuracy of iodine subtraction on virtual non-contrast images [60,61,62].

### 4.3. DECT Contrast Media Reduction

DECT also allows the reduction of the contrast medium dose [63] by using low-energy monoenergetic beams. This is especially useful in patients with a pre-existing renal impairment and a higher risk of contrast-induced nephropathy, as well as elderly patients and patients undergoing routine follow-up [64]. DECT allows synthetic image reconstruction at monochromatic energy levels closer to iodine’s K-edge, where iodine has a substantially higher attenuation than in traditional single-energy acquisition at 120 kVp; this higher attenuation allows a 30% [65,66] reduction in the administered iodine dose for DECT urography when compared to the standard single-energy technique, without compromising attenuation and image quality [67]. Mean DECT attenuation at 50 keV was demonstrated to result in renal vascular and urinary tract attenuation at similar or higher levels than those obtained with the 120 kVp standard acquisition method, with a similar image quality [66].

### 4.4. DECT Stone Composition Analysis

The treatment strategies for stones not only depend on the size and degree of obstruction but also on their chemical composition; for example, uric acid calculi can dissolve in urine at a higher pH and thus an important role is played by urine alkalization, whereas non-invasive treatments, such as extracorporeal shock wave lithotripsy, are less effective in the management of oxalic acid stones. Therefore, the characterization of the chemical composition of ureteral calculi helps in their management. In this regard, the accuracy of DECT is not only helpful for morphological and anatomical evaluation but also for chemical composition.

The attenuation patterns and low and high energy levels are helpful in spectral separation and thus in understanding the chemical composition of the materials, as lighter materials show small differences in attenuation between low and high energy levels. In contrast, those heavier materials show larger differences in attenuation between low and high energy levels.

**Figure 9:**
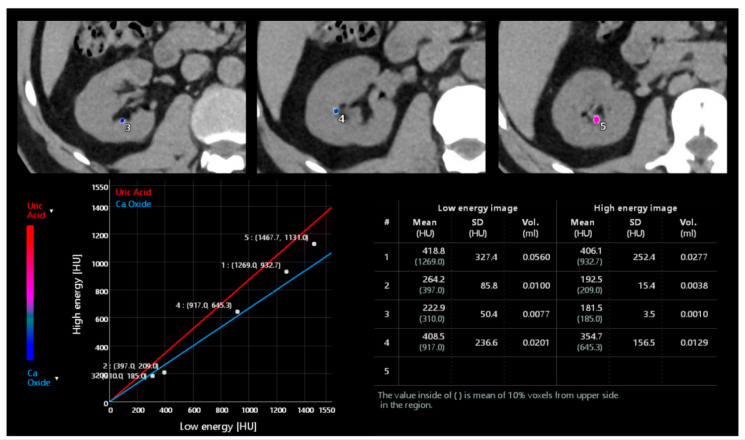
Post-processing analysis of a dual-energy unenhanced kidney acquisition. Different stones can be analyzed to characterize their composition as uric acid, calcium oxide, or mixed composition according to the position of the point related to the straight lines of the two materials. Moreover, an automated calculation of volume and mean Hounsfield Unit is available.

DECT demonstrated high accuracy in the distinction between uric acid calculi from non–uric acid calculi [68,69] and provided information for evaluating stone fragility (Figure 9) [70].

According to attenuation levels, the composition of the stones can also be classified as hydroxyapatite, uric acid, cysteine, oxalic acid, and mixed stones [71]. Accuracy in stone characterization decreases when evaluating calculi < 3–5 mm due to the difficulty in obtaining accurate attenuation values [72].

### 4.5. DECT Iodine Maps

**Figure 10:**
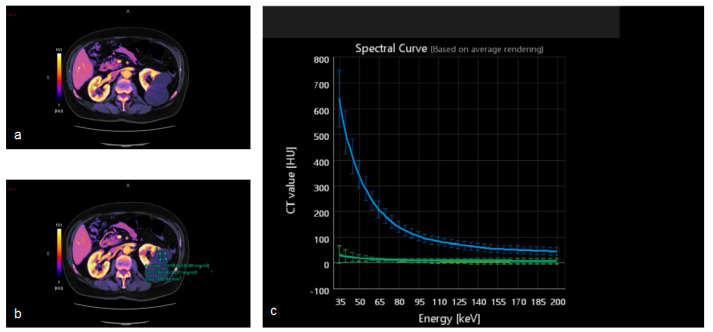
Dual-energy acquisition of a corticomedullary phase. In (**a**) the iodine map shows the absence of iodine within the left renal cysts. To confirm it, a region of interest can be placed in the cyst (**b**) and in the aorta, to obtain the spectral curves. (**c**) The green curve confirms the absent contrast enhancement within the renal cyst, in comparison with the blue curve showing the enhancement within the aortic lumen.

**Figure 11:**
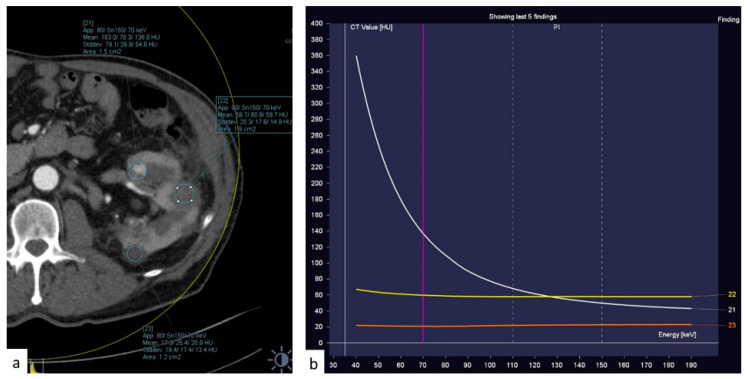
(**a**) Dual-energy acquisition of a corticomedullary phase with visualization of the iodine map. (**b**) The posterior and external renal components do not show iodine contents, whereas the anterior nodulation shows clear iodine content.

**Figure 12:**
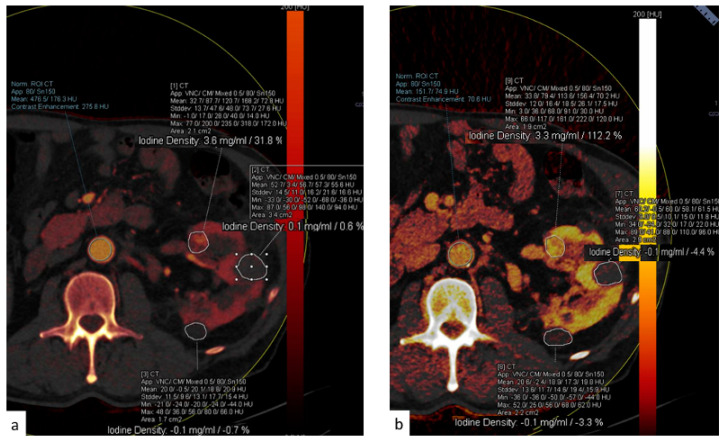
(**a**) The spectral curve of the renal nodules highlighted in Figure 12. (**b**) The yellow and orange lines correspond to the two cysts.

DECT images can also be post-processed to create maps that show the concentration of different components including iodine, fat, and calcium. The construction of iodine maps aids in the identification of tissues with higher or lower iodine concentrations (Figure 10, Figure 11 and Figure 12).

Iodine density measurements have different applications. First, they enable the management of incidentally detected renal masses, allowing their characterization from a single examination and avoiding additional imaging and diagnostic timing delays [73]. Virtual monochromatic images demonstrated better correction of beam hardening artifacts, reducing the degree of pseudo-enhancement in intraparenchymal renal cysts, and allowing for a more confident diagnosis, when compared to standard CT acquisitions [74].

Iodine density measurements and iodine overlay images provide a visual depiction of enhancement that helps in the discrimination between avidly enhancing clear cell renal cell carcinoma and hypoenhancing papillary renal cell carcinoma [75] and is also particularly useful in the assessment of treatment response when using targeted antiangiogenic agents for clear cell renal cell carcinoma [76] and in patients treated with ablation [77].

The advantages of DECT are listed in Table 3.

## 5. Artificial Intelligence

Artificial intelligence models have demonstrated great advances in image analyses [78] with different applications in the radiological field [79]. It can be used for the automatic detection of pathology, for the segmentation of abnormalities [80] and for their characterization (benign versus malignant, and types of neoplasms) [81,82], staging of the disease, risk stratification, and for the prediction of the patients ‘outcome and response to specific treatments to provide tailored management approaches [83].

### 5.1. Computer-Aided Detection

Computer-aided detection (CAD) is a helpful technique in cancer detection [9,84]. A critical step in developing a CAD system is represented by the segmentation of the abnormalities, as it determines the search region for the following steps.

Since urothelial cancer segmentation, particularly in the bladder region, is extremely difficult, researchers’ attention is now concentrated on this issue, and, to our knowledge, no computer-aided detection method for the automatic identification of tumors in the excretory system is currently available.

A computer-aided detection system has been developed and tested to identify exophytic renal lesions on computed tomographic colonography with a 95% sensitivity [85,86]; another study found that gray-level threshold segmentation of the kidneys followed by texture analysis has 85% sensitivity for detecting kidney tumors and no false-positive findings [87].

### 5.2. Segmentation

Accurate segmentation of bladder lesions in CTU is challenging: the bladder can be inhomogeneously filled with contrast medium during the excretory phase, bladder shape varies widely with the level of distension, and the edges of bladder lesions are hardly distinguishable from surrounding soft tissues. Additionally, a hypertrophic prostate protruding into the bladder may be an adjunctive confounding factor. Micro-ultrasound may acquire a role in the urological field in the future, particularly in terms of bladder and prostate cancer detection and infiltration estimation, even if the evidence is still limited at present [88].

When trained with a substantial amount of data, convolutional neural networks demonstrated the ability to categorize medical images, and are used to identify and classify pathological patterns in medical images. They can be trained to recognize patterns inside and outside the bladder and to construct a bladder probability map to assist level set segmentation, with encouraging results in automatic bladder lesion segmentation [80,89,90,91].

### 5.3. Texture Analysis and Radiomics

There is an increasing interest in texture and radiomics analysis as non-invasive tools for the assessment of oncologic and non-oncologic disorders [92,93].

Image texture analyses can identify the differences in the gray scale included in a region of interest [94]: the image of a rough-textured material would have a higher rate of change in the gray-scale value when compared with a smooth-textured material, and the gray-scale values creating the image and the spatial relationships of these values are associated with tissues characteristics, and genetic and other molecular variations when dealing with malignancies [95].

Radiomics is based on the extraction of many quantitative features from medical images [83] by taking advantage of data characterization learning-based algorithms. The characteristics identified through the radiomic analysis cannot be identified by the human eye [96,97,98].

The identification of histological variants of malignancies has a central role in treatment planning [99,100]; however, the visual distinction of different types of bladder malignancies is difficult. Texture features and radiomics analysis showed good accuracy in the cancer typing, with the demonstration of a greater heterogeneous texture in micropapillary carcinoma than in urothelial cancer [101], and potentiality in differentiating histological variants that represent a significant prognostic factor [102].

This tool can aid clinicians in further sub-classifying bladder cancers on routine imaging, with adjustments of the treatment and patient care [103].

Different histological kidney tumors have different gene expression patterns, prognoses, and responses to molecularly targeted therapies, especially in advanced and metastatic diseases [81,104,105]. Percutaneous renal biopsy still represents the gold standard for the histopathological assessment of renal masses, but it is an invasive procedure that is preferentially avoided in elderly patients [81].

Non-invasive biomarkers are needed for a distinction between different malignant lesions [106]. Promising results have been provided by radiomics in the differentiation between clear cell renal cell carcinomas and non-clear cell renal cell carcinomas (papillary and chromophobe renal cell carcinomas), and between epithelioid angiomyolipomas [107] and renal oncocytoma; in this last case this approach avoids the surgical resection of the benign lesion due to a misdiagnosis [108]. In particular, CT radiomics has good performance in classifying pathological renal tumors [109]. The creation of different models including texture features extracted with segmentation, and non-texture features, such as the lesion attenuation value and the absolute enhancement, allowed us to achieve high values of sensibility and specificity in renal lesion differentiation [81].

### 5.4. Tumor Staging and Grading

Another determinant of treatment planning is tumor staging [110].

Non-muscle-invasive and muscle-invasive bladder cancers have significant differences in prognoses and treatment management. Patients with stage T2 to T4 carcinomas of the bladder are recommended for treatment with neoadjuvant chemotherapy.

The local staging and definition of the muscle invasion in bladder cancer are based on cystoscopy and histological evaluation of the biopsied tissues. However, this is an invasive examination and biopsy is dependent both on the operator’s experience and on the sampled portion of the tumor, resulting in wrong staging in up to 25% of cases [111,112]. Repeated procedures could improve the accuracy, at the expense of increased invasiveness. The development of accurate non-invasive methods to assess the local staging is highly desirable.

Artificial intelligence algorithms have been applied to predict the muscle-invasive status of bladder cancers. Different models are available. Deep learning has gained significant attention in recent years; deep-learning models can automatically learn features extracted from radiological images without any need for prior labeling by human experts, reducing the time for manual pre-processing tasks. They can automatically segment pathological tissues and provide phenotypic features of the tissues to enable an accurate characterization.

A proposed deep-learning model based on CT images demonstrated good accuracy in establishing the muscle-invasive status of bladder cancer preoperatively [113].

Texture parameters extracted from urothelial cancer segmentations demonstrated a significant role in the differentiation between both low-grade urothelial cancers from high-grade urothelial cancer and non-muscle-invasive cancer from muscle-invasive on unenhanced, arterial-phase, and venous-phase CT [99]. The combination of morphological characteristics, extracted by an automatic segmentation system, and texture features combined in a machine-learning model showed promising results in the distinction between stages greater than or equal to stage T2 and below stage T2 [114,115].

The grade of clear cell renal carcinoma is usually performed by applying the World Health Organization and International Society of Urological Pathology grading system [116], involving the assessment of nucleolar characteristics or using the Fuhrman grading system [117]. These systems require invasive tissue biopsies and are susceptible to sampling and interobserver errors [118], and a non-invasive, objective tool to assess the whole-tumor grade is still needed to accurately describe the tumor heterogeneity compared to conventional tissue sampling. Tumor segmentation with radiomic features extraction and the creation of machine learning models demonstrated high accuracy in the grading stratification of clear renal cell carcinomas [119,120,121] and chromophobe renal cell carcinomas [109], both in tri-phasic contrast-enhanced CT and in different monochromatic DECT images [122].

### 5.5. Prediction of Treatment Response

One of the main applications of artificial intelligence models is represented by the prediction of response to treatment in oncological patients [123].

In patients affected by bladder cancer with muscular involvement, neoadjuvant chemotherapy before radical cystectomy can improve the resectability of larger neoplasms before radical cystectomy and patients’ survival, and reduce the rate of metastatic disease [124]. However, neoadjuvant chemotherapy has substantial side effects including neutropenia, granulocytopenia, sepsis, mucositis, nausea, and vomiting [125]. It is therefore of pivotal importance to select patients who will respond to these treatments to avoid the toxicity in potentially unresponsive patients and to provide alternative therapies to unresponsive patients [126]; moreover, if a patient can be reliably identified as having a complete response to treatment, the option of organ preservation therapy instead of cystectomy may be considered [127].

No reliable method yet exists to predict the response of individual cases; therefore, there is an increasing interest in the development of computerized artificial intelligence-based predictive models and decision support systems to help the physician in the most appropriate selection of treatment options [128].

Deep learning convolutional neural network systems can be trained to predict the patients who will be responsive to neoadjuvant therapies by correlating imaging features in the pre-treatment and post-treatment CT with histology post cystectomy. Multiple features were identified on the pre- and post-treatment CT images, including shape, size, and texture characteristics which were extracted through lesion segmentation and included in the analysis [128,129].

Immunotherapy is one of the most significant advancements in cancer treatment, although it is only effective in a small number of patients. Because there are currently no biomarkers that can be used to identify individuals who are candidates for this treatment, researchers have turned to artificial intelligence models [130]. Convolutional neural networks and radiomics have thus been used to investigate immunotherapy patients who have progressed after receiving first-line platinum-based chemotherapy, to identify those with a high possibility of responding (complete, partial response, or stable disease), from those who were likely to manifest disease progression; the proposed model demonstrated a 92% accuracy in distinguishing between the two categories of patients [131].

These newly developed tools can assist oncologists in the selection of patients eligible for different treatments [132] to provide personalized medicine.

## 6. Limitations

Although it is a valid and gold standard method for the evaluation of urinary pathologies, CTU has some limitations.

First, the triple phase acquisition protocol, including the non-contrast enhanced phase, the corticomedullary, nephrographic, and excretory phases, is associated with significant radiation exposure [37]. The use of alternative protocols or DECT with virtual unenhanced scan reconstruction may reduce the radiation exposure.

As the identification of urothelial lesions relies on optimal distention and opacification of the excretory system, CTU can be limited by ureteral peristaltic contractions that may impede the complete distention of the ureters. Delayed or suboptimal opacification may be also related to excretory system obstruction or altered renal function, resulting in potential missed urothelial lesions in unopacified segments. Prone acquisitions may improve excretory system distention and opacification but increase the radiation dose.

Moreover, CTU is based on iodinated contrast medium administration, and this fact represents a limitation in patients with impaired renal function. The diffusion of DECT with a reduction of the needed contrast may represent a partial solution to this issue.

## 7. Conclusions

CTU is one of the cornerstone examinations of radiology. A great knowledge of different acquisition protocols, as well as reconstruction algorithms, dual-energy applications, and new perspectives provided by artificial intelligence tools may help radiologists in optimizing everyday practice.

## Figures and Tables

**Table 1:** Clinical indications for Computed Tomography Urography (CTU).

Clinical Indications for CTU
Micro and/or macrohematuria suspicious for urologic malignancy
Staging and follow-up for urothelial malignancy
Iatrogenic or traumatic injuries
Congenital abnormalities
Urinary tract obstruction
Infiltration by pelvic and abdominal tumors
Pre-operative assessment of kidney donors
Post-operative urinary tract anatomy

**Table 2:** Different protocols of contrast medium administration for Computed Tomography Urography.

Technique	Scanning Protocol
Triple phase (conventional single-energy CT)	Non-enhanced phase Intravenous contrast agent injection as a single bolus Corticomedullary phase (optional) 30–40 s after bolus Nephrogenic phase (80–120 s after bolus) Excretory phase (5–15 min after bolus)
Dual-phase split bolus (conventional single-energy CT)	Non-enhanced phase First intravenous contrast agent injection (first bolus) After 5–15 min, a second intravenous contrast agent injection (second bolus) Combined nephrogenic and excretory phase (2–5 min after the second bolus)
Single-phase triple bolus (conventional single-energy CT)	First intravenous contrast agent injection (first bolus) After 5–15 min, a second intravenous contrast agent injection (second bolus) After 100 s, the third intravenous contrast agent injection (third bolus) Combined cortico-medullary, nephrogenic, and excretory phase (25 s after the third bolus)
Single-phase Dual-Energy CT	Single bolus (or split bolus) contrast agent injection Single excretory phase (often 80 and 140 kVp) Postprocessing to generate virtual unenhanced image

**Table 3:** Possible benefits provided by the use of Dual-Energy Computed Tomography (DECT) in the acquisition of Computed Tomography Urography (CTU).

Benefits Provided by DECT
Dose reduction
Reduction of the administered contrast medium
Stone composition analysis
Availability of iodine maps

## Data Availability

Not applicable.
